# Supplementary material for: Influences of Sex, Education, and Country of Birth on Clinical Presentations and Overall Outcomes of Interdisciplinary Pain Rehabilitation in Chronic Pain Patients: A Cohort Study from the Swedish Quality Registry for Pain Rehabilitation (SQRP)
Source: J Clin Med. 2020 Jul 25;9(8):2374. doi: 10.3390/jcm9082374 (PMC7466148; doi:10.3390/jcm9082374)
Supplement: Supplementary file 1 [file jcm-09-02374-s001.pdf]

Journal of
Clinical Medicine

MDPI

Table S1. Changes (mean and SD together with n) between baseline and immediately after IMMRP in the repeated outcomes in women and in men. Group comparison (t-test) and effect size (ES; computed when t-test had p<0.001) are to the far right.

Sex	**Women**			**Men**			Statistics	ES
Variables	n	Mean	SD	N	Mean	SD	p	(Hedges´ g)
**NRS-7d**	10 801	0.89	2.03	3 345	0.95	2.01	0.184	
**HAD-A**	11 270	1.24	3.91	3 504	1.15	3.68	0.212	
**HAD-D**	11 268	1.86	3.85	3 504	1.58	3.67	<0.001	
**MPI-Pain sever**	11 209	0.52	0.98	3 483	0.51	1.02	0.711	
**MPI-Pain interfer**	11 102	0.46	0.92	3 450	0.42	0.91	0.085	
**MPI-control**	11 194	0.59	1.23	3 493	0.52	1.21	0.003	
**MPI-distress**	11 201	0.56	1.38	3 496	0.59	1.28	0.309	
**MPI-SOCSupp**	11 165	−0.19	1.01	3 453	−0.28	0.98	<0.001	0.09
**MPI-punish**	10 087	0.02	1.16	2 967	0.03	1.14	0.666	
**MPI-protect**	10 041	−0.11	1.06	2 958	−0.15	0.99	0.070	
**MPI-distract**	10 081	0.02	1.02	2 967	0.00	0.98	0.179	
**MPI-GAI**	11 196	0.19	0.72	3 480	0.20	0.78	0.596	
**EQ-5D-index**	10 614	0.13	0.33	3 375	0.14	0.33	0.404	
**EQ-VAS**	10 459	10.06	22.15	3 318	8.83	21.59	0.005	
**sf36-pf**	10 834	4.78	15.99	3 419	5.32	16.50	0.091	
**sf36-rp**	10 611	10.11	33.63	3 334	9.36	33.58	0.264	
**sf36-bp**	10 847	8.71	16.62	3 421	8.26	16.85	0.170	
**sf36-gh**	10 638	5.30	17.23	3 350	4.04	17.04	<0.001	0.07
**sf36-vt**	10 805	12.08	21.79	3 401	10.53	21.68	<0.001	0.07
**sf36-sf**	10 827	7.75	25.59	3 402	7.29	24.51	0.355	
**sf36-re**	10 442	8.44	47.58	3 259	8.21	45.78	0.812	
**sf36-mh**	10 796	7.62	20.02	3 398	7.22	19.52	0.313	

* = small effect ES, ** = medium effect ES, *** large effect ES; NRS-7d = pain intensity previous 7 days; HAD=Hospital Anxiety and Depression Scale; HAD-A=subscale anxiety; HAD-D=subscale depression; MPI=Multidimensional Pain inventory: MPI-Pain-sever=subscale pain severity; MPI-Pain-interfer=subscale pain related Interference; MPI-control=subscale life control; MPI-distress=subscale affective distress; MPI-SOCSupp=subscale social support; MPI-punish=subscale punishing responses; MPI-protect=subscale solicitous responses; MPI-distract=subscale distracting responses; MPI-GAI=subscale General Activity Index; EQ=European Quality of Life instrument; EQ-5D-index=index based om five dimensions; EQ-VAS=self-estimation of health; sf36=Short Form Health Survey; sf36-pf=physical functioning; sf36-rp=role limitations due to physical functioning; sf36-bp=bodily pain; sf36-gh=general health; sf36-vt=vitality; sf36-sf=social functioning; sf36-re=role limitations due to emotional problems; sf36-mh=mental health

Table S2. Changes (mean and SD together with n) between baseline and 12-month follow-up in the repeated outcomes in women and in men. Group comparison (t-test) and effect size (ES; computed when t-test had p<0.001) are to the far right.

Sex	**Women**			**Men**			Statistics	ES
Variables	n	Mean	SD	n	Mean	SD	t-test	(Hedges´ g)
**NRS-7d**	6 594	1.05	2.20	1 974	1.08	2.29	0.661	
**HAD-A**	6 828	1.46	4.09	2 037	0.98	4.02	<0.001	0.12
**HAD-D**	6 830	1.58	4.07	2 035	0.98	4.14	<0.001	0.15
**MPI-Pain sever**	6 862	0.66	1.15	2 042	0.64	1.24	0.516	
**MPI-Pain interfer**	6 811	0.62	1.14	2 018	0.57	1.14	0.049	
**MPI-control**	6 834	0.53	1.28	2 037	0.44	1.29	0.007	
**MPI-distress**	6 848	0.51	1.44	2 041	0.46	1.41	0.198	
**MPI-SOCSupp**	6 813	−0.39	1.16	2 017	−0.46	1.14	0.028	
**MPI-punish**	6 103	0.01	1.24	1 721	−0.01	1.20	0.519	
**MPI-protect**	6 075	−0.17	1.19	1 709	−0.22	1.11	0.177	
**MPI-distract**	6 092	−0.06	1.10	1 719	−0.09	1.01	0.379	
**MPI-GAI**	6 832	0.17	0.80	2 027	0.14	0.85	0.056	
**EQ-5D-index**	6 701	0.17	0.36	2 143	0.19	0.36	0.027	
**EQ-VAS**	6 541	11.38	23.85	2 066	10.04	23.87	0.026	
**sf36-pf**	6 500	6.79	18.36	1 959	6.23	19.12	0.235	
**sf36-rp**	6 387	14.43	37.44	1 914	15.50	38.52	0.272	
**sf36-bp**	6 498	10.81	19.06	1 960	10.81	20.16	0.996	
**sf36-gh**	6 412	5.22	19.29	1 930	3.23	18.84	<0.001	0.10
**sf36-vt**	6 489	9.75	23.01	1 952	8.46	22.08	0.028	
**sf36-sf**	6 503	9.23	27.19	1 956	6.98	26.71	0.001	
**sf36-re**	6 281	10.82	49.51	1 878	11.21	49.76	0.766	
**sf36-mh**	6 485	6.76	21.42	1 950	5.03	20.94	0.002	

* = small effect ES, ** = medium effect ES, *** large effect ES; NRS-7d = pain intensity previous 7 days; HAD=Hospital Anxiety and Depression Scale; HAD-A=subscale anxiety; HAD-D=subscale depression; MPI=Multidimensional Pain inventory: MPI-Pain-sever=subscale pain severity; MPI-Pain-interfer=subscale pain related Interference; MPI-control=subscale life control; MPI-distress=subscale affective distress; MPI-SOCSupp=subscale social support; MPI-punish=subscale punishing responses; MPI-protect=subscale solicitous responses; MPI-distract=subscale distracting responses; MPI-GAI=subscale General Activity Index; EQ=European Quality of Life instrument; EQ-5D-index=index based om five dimensions; EQ-VAS=self-estimation of health; sf36=Short Form Health Survey; sf36-pf=physical functioning; sf36-rp=role limitations due to physical functioning; sf36-bp=bodily pain; sf36-gh=general health; sf36-vt=vitality; sf36-sf=social functioning; sf36-re=role limitations due to emotional problems; sf36-mh=mental health

Table S3. Changes (mean and SD together with n) between baseline and immediately after IMMRP in the repeated outcomes for education level. Group comparison (ANOVA with post hoc tests) and effect size (ES for Elementary school vs. University; computed when ANOVA had p<0.001) are to the far right.

Education level	Elementary school			Upper secondary school			University			ANOVA		ES
Variables	N	Mean	SD	N	Mean	SD	N	Mean	SD	p-value	post-hoc	(Hedges´ g)
**NRS-7d**	2655	0.85	1.99	7808	0.88	2.01	3505	0.99	2.07	0.008	NA	
**HAD-A**	2781	1.03	3.82	8107	1.20	3.88	3651	1.42	3.79	<0.001	Esc=USS, other different	0.10
**HAD-D**	2782	1.71	3.82	8105	1.78	3.81	3652	1.91	3.79	0.098	NA	
**MPI-Pain sever**	2748	0.45	0.97	8093	0.52	0.98	3624	0.55	1.01	<0.001	USS=U, other different	0.10
**MPI-Pain interfer**	2714	0.41	0.90	8029	0.44	0.90	3586	0.49	0.95	<0.001	Esc=USS, other different	0.09
**MPI-control**	2744	0.51	1.25	8102	0.57	1.23	3613	0.62	1.20	0.001	NA	
**MPI-distress**	2752	0.53	1.36	8101	0.57	1.37	3616	0.61	1.33	0.057	NA	
**MPI-SOCSupp**	2736	-0.23	0.99	8060	-0.21	1.02	3595	-0.21	0.98	0.717	NA	
**MPI-punish**	2417	0.02	1.19	7198	0.01	1.17	3254	0.03	1.10	0.594	NA	
**MPI-protect**	2404	-0.14	1.08	7171	-0.11	1.08	3239	-0.15	0.95	0.115	NA	
**MPI-distract**	2419	0.01	1.04	7196	0.03	1.02	3247	-0.01	0.95	0.210	NA	
**MPI-GAI**	2750	0.17	0.74	8084	0.20	0.74	3615	0.19	0.72	0.304	NA	
**EQ-5D-index**	2605	0.11	0.33	7644	0.13	0.33	3501	0.14	0.33	0.005	NA	
**EQ-VAS**	2553	8.41	23.24	7524	9.73	22.06	3475	10.69	20.88	<0.001	USS=U, other different	0.10
**sf36-pf**	2689	4.70	16.73	7800	4.76	16.00	3555	5.32	15.79	0.182	NA	
**sf36-rp**	2592	7.77	32.86	7646	9.91	33.49	3504	11.40	34.14	<0.001	USS=U, other different	0.11

**sf36-bp**	2688	7.58	16.52	7810	8.55	16.53	3560	9.42	17.00	<0.001	all different	
**sf36-gh**	2620	3.95	17.08	7675	4.81	17.22	3493	6.15	17.15	<0.001	Esc=USS, other different	0.13
**sf36-vt**	2673	10.87	21.63	7779	11.59	21.65	3546	12.64	22.05	0.005	NA	
**sf36-sf**	2680	5.32	25.70	7786	7.36	25.06	3557	10.03	25.35	<0.001	all different	0.18
**sf36-re**	2541	7.23	47.06	7505	8.25	47.07	3462	9.36	47.32	0.219	NA	
**sf36-mh**	2668	7.20	20.38	7775	7.59	19.90	3545	7.69	19.42	0.599	NA	

* = small effect ES, ** = medium effect ES, *** large effect ES; Esc=Elementary School; USS=upper secondary school; U=University; NRS-7d = pain intensity previous 7 days; HAD=Hospital Anxiety and Depression Scale; HAD-A=subscale anxiety; HAD-D=subscale depression; MPI=Multidimensional Pain inventory: MPI-Pain-sever=subscale pain severity; MPI-Pain-interfer=subscale pain related Interference; MPI-control=subscale life control; MPI-distress=subscale affective distress; MPI-SOCSupp=subscale social support; MPI-punish=subscale punishing responses; MPI-protect=subscale solicitous responses; MPI-distract=subscale distracting responses; MPI-GAI=subscale General Activity Index; EQ=European Quality of Life instrument; EQ-5D-index=index based om five dimensions; EQ-VAS=self-estimation of health; sf36=Short Form Health Survey; sf36-pf=physical functioning; sf36-rp=role limitations due to physical functioning; sf36-bp=bodily pain; sf36-gh=general health; sf36-vt=vitality; sf36-sf=social functioning; sf36-re=role limitations due to emotional problems; sf36-mh=mental health

Table S4. Changes (mean and SD together with n) between baseline and 12-month follow-up in the repeated outcomes for education level. Group comparison (ANOVA with post hoc tests) and effect size (ES for Elementary school vs. University); computed when ANOVA had p<0.001) are to the far right.

Education level	Elementary school			Upper secondary school			University			ANOVA		ES
Variables	N	Mean	SD	N	Mean	SD	N	Mean	SD	p-value	post-hoc	(Hedges´ g)
**NRS-7d**	1613	0.89	2.15	4705	1.05	2.21	2139	1.19	2.29	<0.001	all different	0.13
**HAD-A**	1665	1.20	4.11	4819	1.32	4.07	2231	1.57	4.02	0.011	NA	
**HAD-D**	1666	1.20	4.17	4819	1.43	4.06	2231	1.71	4.06	<0.001	Esc=USS, other different	0.12
**MPI-Pain sever**	1672	0.51	1.14	4851	0.65	1.16	2233	0.77	1.18	<0.001	all different	0.22*
**MPI-Pain interfer**	1661	0.48	1.07	4806	0.61	1.14	2217	0.72	1.19	<0.001	all different	0.21*

**MPI-control**	1668	0.41	1.28	4830	0.51	1.30	2225	0.58	1.24	<0.001	USS=U, other different	0.14
**MPI-distress**	1669	0.38	1.42	4839	0.50	1.44	2234	0.58	1.40	<0.001	USS=U, other different	0.14
**MPI-SOCSupp**	1658	-0.36	1.19	4819	-0.40	1.15	2207	-0.46	1.13	0.020	NA	
**MPI-punish**	1436	-0.03	1.25	4278	0.01	1.25	1985	0.02	1.17	0.485	NA	
**MPI-protect**	1428	-0.19	1.23	4260	-0.18	1.19	1973	-0.19	1.09	0.960	NA	
**MPI-distract**	1433	-0.05	1.10	4275	-0.06	1.08	1979	-0.10	1.06	0.200	NA	
**MPI-GAI**	1668	0.13	0.81	4821	0.16	0.80	2224	0.22	0.81	0.001	NA	
**EQ-5D-index**	1662	0.15	0.35	4747	0.17	0.36	2264	0.20	0.36	<0.001	all different	0.14
**EQ-VAS**	1617	9.08	24.91	4619	10.92	23.96	2209	12.60	22.60	<0.001	all different	0.15
**sf36-pf**	1590	4.01	18.86	4569	6.84	18.37	2169	8.03	18.45	<0.001	all different	0.22*
**sf36-rp**	1539	11.42	36.68	4489	14.92	37.71	2143	16.37	37.90	<0.001	USS=U, other different	0.13
**sf36-bp**	1594	8.62	18.33	4570	10.82	19.23	2162	12.32	19.93	<0.001	all different	0.19
**sf36-gh**	1561	2.76	18.98	4512	4.83	19.17	2140	6.13	19.31	<0.001	all different	0.18
**sf36-vt**	1583	8.13	22.38	4563	9.36	22.57	2163	10.54	23.44	0.006	NA	
**sf36-sf**	1592	5.41	26.97	4572	8.20	26.66	2163	12.29	27.68	<0.001	all different	0.25*
**sf36-re**	1500	8.39	47.96	4410	11.55	49.45	2122	11.47	50.87	0.087	NA	
**sf36-mh**	1582	5.37	21.61	4559	6.45	21.30	2163	7.05	20.93	0.056	NA	

* = small effect ES, ** = medium effect ES, *** large effect ES; Esc=Elementary School; USS=upper secondary school; U=University; NRS-7d = pain intensity previous 7 days; HAD=Hospital Anxiety and Depression Scale; HAD-A=subscale anxiety; HAD-D=subscale depression; MPI=Multidimensional Pain inventory: MPI-Pain-sever=subscale pain severity; MPI-Pain-interfer=subscale pain related Interference; MPI-control=subscale life control; MPI-distress=subscale affective distress; MPI-SOCSupp=subscale social support; MPI-punish=subscale punishing responses; MPI-protect=subscale solicitous responses; MPI-distract=subscale distracting responses;

MPI-GAI=subscale General Activity Index; EQ=European Quality of Life instrument; EQ-5D-index=index based om five dimensions; EQ-VAS=self-estimation of health; sf36=Short Form Health Survey; sf36-pf=physical functioning; sf36-rp=role limitations due to physical functioning; sf36-bp=bodily pain; sf36-gh=general health; sf36-vt=vitality; sf36-sf=social functioning; sf36-re=role limitations due to emotional problems; sf36-mh=mental health

Table S5. Changes (mean and SD together with n) between baseline and immediately after IMMRP in the repeated outcomes in patients born in Europe vs. patients born outside Europe. Group comparison (t-test) and effect size (ES; computed when t-test had p<0.001) are to the far right.

Country of birth	Europe			Outside Europe			Statistics	ES
Variables	N	Mean	SD	N	Mean	SD	p-value	(Hedges´ g)
**NRS-7d**	12 631	0.89	2.03	1 444	1.05	1.99	0.005	
**HAD-A**	13 098	1.19	3.81	1 546	1.53	4.17	0.001	
**HAD-D**	13 093	1.82	3.77	1 550	1.61	4.16	0.042	
**MPI-Pain sever**	13 061	0.52	0.99	1 509	0.46	0.95	0.035	
**MPI-Pain interfer**	12 964	0.45	0.91	1 472	0.38	0.93	0.003	
**MPI-control**	13 069	0.57	1.22	1 499	0.55	1.29	0.496	
**MPI-distress**	13 070	0.57	1.35	1 508	0.55	1.37	0.609	
**MPI-SOCSupp**	13 000	-0.22	1.00	1 497	-0.17	1.08	0.108	
**MPI-punish**	11 667	0.03	1.13	1 293	-0.05	1.37	0.014	
**MPI-protect**	11 625	-0.12	1.03	1 280	-0.17	1.18	0.087	
**MPI-distract**	11 658	0.02	0.98	1 296	-0.01	1.24	0.372	
**MPI-GAI**	13 062	0.19	0.69	1 494	0.21	1.02	0.186	
**EQ-5D-index**	12 359	0.13	0.33	1 486	0.13	0.35	0.754	
**EQ-VAS**	12 229	10.01	21.80	1 419	7.39	23.63	<0.001	0.12
**sf36-pf**	12 662	4.95	15.79	1 483	4.31	18.54	0.144	
**sf36-rp**	12 476	10.33	33.53	1 364	5.87	34.11	<0.001	0.13
**sf36-bp**	12 680	8.57	16.54	1 480	8.89	17.67	0.486	
**sf36-gh**	12 475	5.15	17.14	1 410	3.53	17.63	0.001	
**sf36-vt**	12 633	11.95	21.59	1 467	9.74	23.19	<0.001	0.10
**sf36-sf**	12 654	7.70	25.25	1 467	6.88	25.93	0.242	
**sf36-re**	12 296	8.33	47.26	1 306	8.49	46.13	0.906	
**sf36-mh**	12 625	7.56	19.60	1 464	7.16	22.23	0.464	

* = small effect ES, ** = medium effect ES, *** large effect ES; NRS-7d = pain intensity previous 7 days; HAD=Hospital Anxiety and Depression Scale; HAD-A=subscale anxiety; HAD-D=subscale depression; MPI=Multidimensional Pain inventory: MPI-Pain-sever=subscale pain severity; MPI-Pain-interfer=subscale pain related Interference; MPI-control=subscale life control; MPI-distress=subscale affective distress; MPI-SOCSupp=subscale social support; MPI-punish=subscale punishing responses; MPI-protect=subscale solicitous responses; MPI-distract=subscale distracting responses; MPI-GAI=subscale General Activity Index; EQ=European Quality of Life instrument; EQ-5D-index=index based om five dimensions; EQ-VAS=self-estimation of health; sf36=Short Form Health Survey; sf36-pf=physical functioning; sf36-rp=role limitations due to physical functioning; sf36-bp=bodily pain; sf36-gh=general health; sf36-vt=vitality; sf36-sf=social functioning; sf36-re=role limitations due to emotional problems; sf36-mh=mental health

Table S6. Changes (mean and SD together with n) between baseline and 12-month follow-up in the repeated outcomes in patients born in Europe vs. patients born outside Europe. Group comparison (t-test) and effect size (ES; computed when t-test had p<0.001) are to the far right.

Country of birth	Europe			Outside Europe			Statistics	ES
Variables	N	Mean	SD	N	Mean	SD	p-value	(Hedges´ g)
**NRS-7d**	7733	1.05	2.23	799	1.07	2.18	0.795	
**HAD-A**	7963	1.35	4.04	830	1.39	4.49	0.777	
**HAD-D**	7961	1.51	4.04	833	0.80	4.56	<0.001	0.17
**MPI-Pain sever**	8013	0.66	1.17	824	0.56	1.12	0.018	
**MPI-Pain interfer**	7959	0.62	1.14	806	0.47	1.15	<0.001	0.13
**MPI-control**	7992	0.52	1.27	812	0.39	1.38	0.007	
**MPI-distress**	8005	0.50	1.43	818	0.38	1.43	0.018	
**MPI-SOCSupp**	7948	-0.41	1.15	816	-0.33	1.18	0.041	
**MPI-punish**	7080	0.02	1.21	691	-0.12	1.41	0.007	
**MPI-protect**	7047	-0.18	1.17	684	-0.20	1.21	0.645	
**MPI-distract**	7070	-0.06	1.06	689	-0.16	1.24	0.018	
**MPI-GAI**	7986	0.17	0.78	806	0.14	1.03	0.338	
**EQ-5D-index**	7850	0.17	0.36	905	0.20	0.37	0.048	
**EQ-VAS**	7669	11.25	23.84	854	8.74	23.80	0.004	
**sf36-pf**	7619	6.77	18.32	783	5.38	20.42	0.045	
**sf36-rp**	7520	15.13	37.65	724	9.19	37.40	<0.001	0.16
**sf36-bp**	7617	10.75	19.22	783	10.98	19.86	0.751	
**sf36-gh**	7531	4.95	19.15	757	2.75	19.61	0.003	
**sf36-vt**	7604	9.58	22.71	779	7.75	23.50	0.032	
**sf36-sf**	7622	8.69	27.01	780	8.30	27.73	0.700	
**sf36-re**	7432	10.86	49.67	673	10.62	48.62	0.905	
**sf36-mh**	7602	6.41	21.06	776	5.57	23.65	0.296	

* = small effect ES, ** = medium effect ES, *** large effect ES; NRS-7d = pain intensity previous 7 days; HAD=Hospital Anxiety and Depression Scale; HAD-A=subscale anxiety; HAD-D=subscale depression; MPI=Multidimensional Pain inventory: MPI-Pain-sever=subscale pain severity; MPI-Pain-interfer=subscale pain related Interference; MPI-control=subscale life control; MPI-distress=subscale affective distress; MPI-SOCSupp=subscale social support; MPI-punish=subscale punishing responses; MPI-protect=subscale solicitous responses; MPI-distract=subscale distracting responses; MPI-GAI=subscale General Activity Index; EQ=European Quality of Life instrument; EQ-5D-index=index based om five dimensions; EQ-VAS=self-estimation of health; sf36=Short Form Health Survey; sf36-pf=physical functioning; sf36-rp=role limitations due to physical functioning; sf36-bp=bodily pain; sf36-gh=general health; sf36-vt=vitality; sf36-sf=social functioning; sf36-re=role limitations due to emotional problems; sf36-mh=mental health
